# Improve Soybean Variety Selection Accuracy Using UAV-Based High-Throughput Phenotyping Technology

**DOI:** 10.3389/fpls.2021.768742

**Published:** 2022-01-11

**Authors:** Jing Zhou, Eduardo Beche, Caio Canella Vieira, Dennis Yungbluth, Jianfeng Zhou, Andrew Scaboo, Pengyin Chen

**Affiliations:** ^1^Division of Plant Science and Technology, University of Missouri, Columbia, MO, United States; ^2^Fisher Delta Research Center, University of Missouri, Portageville, MO, United States

**Keywords:** high-throughput phenotyping, soybean breeding, machine learning, variety selection, Unmanned Aerial Vehicle (UAV)

## Abstract

The efficiency of crop breeding programs is evaluated by the genetic gain of a primary trait of interest, e.g., yield, achieved in 1 year through artificial selection of advanced breeding materials. Conventional breeding programs select superior genotypes using the primary trait (yield) based on combine harvesters, which is labor-intensive and often unfeasible for single-row progeny trials (PTs) due to their large population, complex genetic behavior, and high genotype-environment interaction. The goal of this study was to investigate the performance of selecting superior soybean breeding lines using image-based secondary traits by comparing them with the selection of breeders. A total of 11,473 progeny rows (PT) were planted in 2018, of which 1,773 genotypes were selected for the preliminary yield trial (PYT) in 2019, and 238 genotypes advanced for the advanced yield trial (AYT) in 2020. Six agronomic traits were manually measured in both PYT and AYT trials. A UAV-based multispectral imaging system was used to collect aerial images at 30 m above ground every 2 weeks over the growing seasons. A group of image features was extracted to develop the secondary crop traits for selection. Results show that the soybean seed yield of the selected genotypes by breeders was significantly higher than that of the non-selected ones in both yield trials, indicating the superiority of the breeder's selection for advancing soybean yield. A least absolute shrinkage and selection operator model was used to select soybean lines with image features and identified 71 and 76% of the selection of breeders for the PT and PYT. The model-based selections had a significantly higher average yield than the selection of a breeder. The soybean yield selected by the model in PT and PYT was 4 and 5% higher than those selected by breeders, which indicates that the UAV-based high-throughput phenotyping system is promising in selecting high-yield soybean genotypes.

## Introduction

Crop breeding is the art and science of improving crop traits to produce desired characteristics (Sleper and Poehlman, 2006). The goal of crop breeding is to improve genetic gains, which is the increase in the performance of a population achieved in 1 year through artificial selection (Fehr, 1991). From 10,000 years ago, plants with preferable traits have been domesticated and selected by humans for increased production and environmental adaptability (Ahmar et al., 2020). Since the early 1900's, crop breeding has been systemized with the introduction of Mendelian laws, followed by cross-breeding and hybrid breeding that are referred to as conventional breeding strategies (Ahmar et al., 2020). Conventional breeding programs still primarily make selection decisions through phenotypic observations that are time-consuming, labor-intensive, and subjective to the experience of breeders (Araus et al., 2018). For example, the development of a new soybean cultivar can take up to 8 years, given the many phases of crossing, selection, and field trials in multiple environments (Ahmar et al., 2020; Vieira et al., 2021). Genetic gains can be increased by enhancing selection intensity, shortening the breeding cycle, ensuring suitable genetic variation in the population, and obtaining accurate estimates of the genetic values (Xu et al., 2017; Araus et al., 2018; Moreira et al., 2019). The selection intensity (or population size) is limited by the phenotyping capacity to measure key agronomic traits of breeding materials and selection accuracy by the lack of objective and efficient phenotyping tools.

Thanks to the advances in high-throughput genotyping and sequencing technologies, molecular breeding strategies have been developed to shorten conventional breeding cycles and increase selection accuracy with the aid of genetic markers (Xu et al., 2017). Two commonly used methods, i.e., marker-assisted selection and genomic selection that rely on marker-trait associations, are routinely applied in modern plant breeding programs (Arruda et al., 2016). Despite genomics and technical advances, identifying plants with desirable traits is the ultimate and most important goal in breeding pipelines (Ahmar et al., 2020). However, it is well-acknowledged in the breeding community that the phenotyping efficiency is still a bottleneck in crop breeding (Araus and Cairns, 2014; Araus et al., 2018). In addition, the lack of accurate phenotypic data has led to poor associations between phenotypes and gene/QTL, limiting the genome-based selection accuracy (Mammadov et al., 2012).

Soybean breeding programs include intensive field trials starting from progeny trials (PTs), where a large number of genotypes (e.g., 30,000) are planted in non-replicated one-row breeding plots due to limited resources, such as seed availability, land, and cost (Orf et al., 2004). In conventional breeding programs, plant traits in PTs are manually collected, which requires a large investment of resources, and data collected are inaccurate due to the border effects of single rows and subjective observations (Moreira et al., 2019). Therefore, critical soybean traits, such as yield and maturity group (MG), are not feasible to collect for all entries. A certain percentage of them will be selected by visual examinations (by experienced breeders) for the advancement in the following yield trials (YTs). In YTs, soybean lines are often planted in four-row plots with two center rows as effective rows while two side rows as buffer rows. Key traits in YTs are manually measured and recorded for each plot and the best performing lines are selected. This procedure will be repeated in many environments for 2–3 years. The massive field phenotyping workload represents a bottleneck in conventional breeding, marker-assisted selection, or genomic selection.

High-throughput phenotyping (HTP) has demonstrated the ability to efficiently acquire sensory data from plants leveraging diverse kinds of spectral sensors, such as high-resolution digital, multi-/hyperspectral, infrared thermal, and fluorescence cameras as well as Lidar (light detection and ranging) devices. Advances in statistical learning theories and application programming interface packages for machine learning (ML) have enabled data assimilation and feature identification for plant phenotyping. ML approaches play key roles in plant HTP, such as detecting corn kernels using convolutional neural networks (CNN) and digital red-green-blue images (Khaki et al., 2020) and identifying soybean flowers and seedpods using region-based CNN (RCNN) (Pratama et al., 2020). ML approaches have also been used to predict plant traits, such as yield by CNN (Zhou et al., 2021b), maturity date by partial least square regression (Zhou et al., 2019) and stress responses by artificial neural networks, decision tree models, linear discriminant analysis, and support vector machine (Bai et al., 2018; Zhou et al., 2020, 2021a).

Agronomic traits developed by HTP systems have been used as secondary traits in the selection of superior soybean breeding lines, which may decrease the population size tested in field conditions and increase selection intensity (Richards, 2000). Secondary traits can be measured at earlier growth stages or generations, which may accelerate the selection procedure and eventually shorten the breeding circles (Moreira et al., 2019). Most importantly, HTP systems can quantify crop traits in PTs that are not able to be collected efficiently in conventional breeding programs (e.g., estimated yield and vegetation indices), and provide data-driven references to select superior progeny rows by learning from the selections of a breeder. To the best of our knowledge, no studies have been conducted to evaluate the performance of secondary traits from HTP systems in selecting superior genotypes from PTs to advance yield.

Motivated by the need to improve the selection accuracy and intensity in soybean breeding, the goal of this study was to evaluate the performance in selecting superior soybean progeny rows using UAV-based image features as secondary traits. There were three objectives to achieve this goal: (1) modeling breeder's selection (selected/non-selected) using manually measured crop traits (flower color, pubescence color, plant height, lodging, maturity date, and yield) to quantify weights of the traits on the final selections, (2) determining the growth stages when the image features are effective in making selections, and (3) evaluating the performance of model selections by comparing their yield performances with breeder's selection.

## Materials and Methods

### Plant Material and Field Experiment

The field experiments were conducted from 2018 to 2020, including a PT in 2018, a preliminary yield trial (PYT) in 2019, and an advanced yield trial (AYT) in 2020. The PT included 11,473 F_4:5_ soybean progeny rows and was conducted in a 3.64 ha field at the Greenley Research Center of the University of Missouri (MU), Novelty, Missouri. The progeny rows were planted without replicates in one-row plots of 2.59 m in length with 0.76 m row spacing. The progeny rows were planted using an Almaco cone plot planter (Almaco, Nevada, Iowa) on May 29, 2018.

A group of 1,860 breeding lines was selected from the PT based on overall yield potential and favorable agronomic traits at maturity, among which 1,773 were advanced to the PYT in 2019 and were planted without replicates in four-row plots with a row length of 3.6 m and a row spacing of 0.8 m. The PYT plots were planted on June 3, 2019, at the Bay Farm Research Facility, Columbia, Missouri. The 2020 AYT consisted of 238 breeding lines selected from the 2019 PYT based on multi-environment yield performance and favorable agronomic traits and were planted in a randomized complete block design with two replicates at the Bay Farm Research Facility, Columbia, Missouri, on June 2, 2020. Plot size, row length, and row spacing for AYT were the same as PYT. In PT, PYT, and AYT, replicated checks for MG III and MG IV were planted over the field as maturity references.

### Manually Measured Agronomic Traits

Six agronomic traits were manually measured for the four-row soybean plots in the PYT and AYT, including flower color, pubescence color, plant height, lodging, maturity date, and grain yield. Soybean flower color was classified into three categories [i.e., purple, white, segregating (mixed colors)], and pubescence color into four categories (i.e., tawny, light tawny, gray, and segregating). All color information was recorded by experienced breeders or research staff. Plant height was measured as the average height from ground level to the top node of the plants in the two center rows of each plot at the maturity stage (R8). Lodging was visually rated on a scale of 1–5 (i.e., 1 = all plants erect; 2 = 5–25% of the plants prostrate; 3 = 25–50% of the plants prostrate; 4 = 50–80% of the plants prostrate; and 5 = all plants prostrate). Plant maturity was visually determined and recorded as the number of days after September 1 (day 1) when 95% of the pods in the two center rows of each plot achieved mature pod color. The total seed weight of the central two rows of each plot was measured by the plot combine Almaco R1 or Almaco SPC-40 (Almaco, Nevada, Iowa). The final grain yield was converted to kg·ha^−1^ on a 13% moisture basis (Beche et al., 2020).

### UAV Data Collection

Imagery data were acquired using a UAV imaging system consisting of a UAV platform (model: DJI Matrice 600 Pro, DJI, Shenzhen, Guangdong, China) and a five-band multispectral (Blue-Green-Red-RedEdge-NIR) camera (RedEdge-M, MicaSense, Seattle, WA, USA). The multispectral camera has an image resolution of 1,280 × 960 pixels and was configured to take time-lapse images at one frame per second (1 fps). The spectral camera was calibrated using a Calibrated Reflectance Panel (Micasense) following the procedure described in Zhou et al. (2019). The multispectral camera has a Global Navigation Satellite System (GNSS) receiver unit embedded and preprogrammed to provide geo-referencing information for each image in exchangeable image file format. Before each flight, a calibration reflectance panel (CRP) was imaged by holding the camera at about 1 m above the CRP and looking vertically in an open area to avoid the shadow.

Data were collected four times [75, 90, 108, and 121 days after planting (DAPs)] in the 2018 PT, six times (45, 57, 74, 87, 109, and 120 DAPs) in the 2019 PYT, and six times (51, 64, 78, 94, 107, and 120 DAPs) in the 2020 AYT. The UAV imaging system flew at 30-m-above ground level with the camera in nadir view for all data collections, resulting in a ground sampling distance of 20.8 mm·pixel^−1^ for the multispectral images. Before each flight, the flight speed was set to 7 km·h^−1^, and the flight paths were designed to ensure a forward overlap of ≥ 70% and a side overlap of ≥ 65% for all images using the flight control App Autopilot (Hangar Technology, Austin, TX, USA).

**Figure 1:**
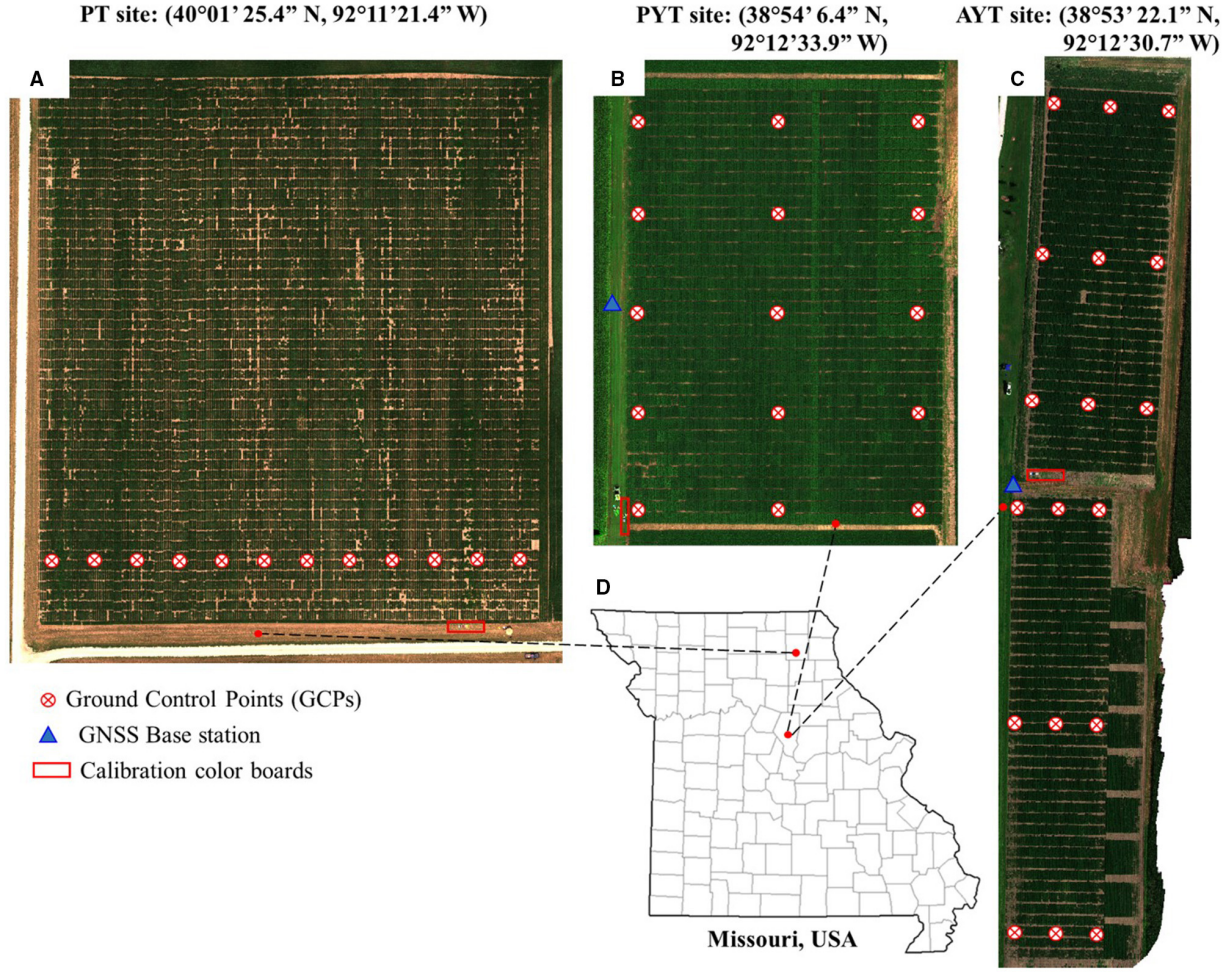
Locations of field trials. **(A–C)** are fields and distribution of ground control points (GCPs) at the progeny trial (PT) in 2018, the preliminary yield trial (PYT) in 2019, and the advanced yield trial (AYT) in 2020, respectively. **(D)** Geographical locations of the fields.

About 12, 15, and 18 ground control points (GCPs) were placed evenly for each data collection in the PT, PYT, and AYT fields, as shown in Figure 1. The GCP was made of a 30 × 30 cm wood square covered with a cross-patterned vinyl sheet that was mounted on the top of a 1.1 m plastic fence post. The GNSS coordinates of the GCPs were obtained using a Real-Time Kinematic (RTK) GNSS positioning system (Reach RS+, Emlid, St. Petersburg, Russia) that consists of a base station and a rover receiver. The base station was mounted on a tribrach that was fixed in an open area in the fields to ensure sufficient satellite reception for each data collection. The base position with an averaged single accuracy (~2.5 m) was obtained for each field by accumulating its GNSS coordinates for 5 min (Emlid, 2021). The base station was maintained stationary and horizontally during every flight and its position was fixed over a growing season. The rover receiver was mounted on a monopod that was placed vertically in the holes after the GCPs were pulled out. The GNSS coordinate of each GCP was recorded after accumulating for 10 s using a manufacturer-developed App ReachView.

### Image Processing

**Figure 2:**
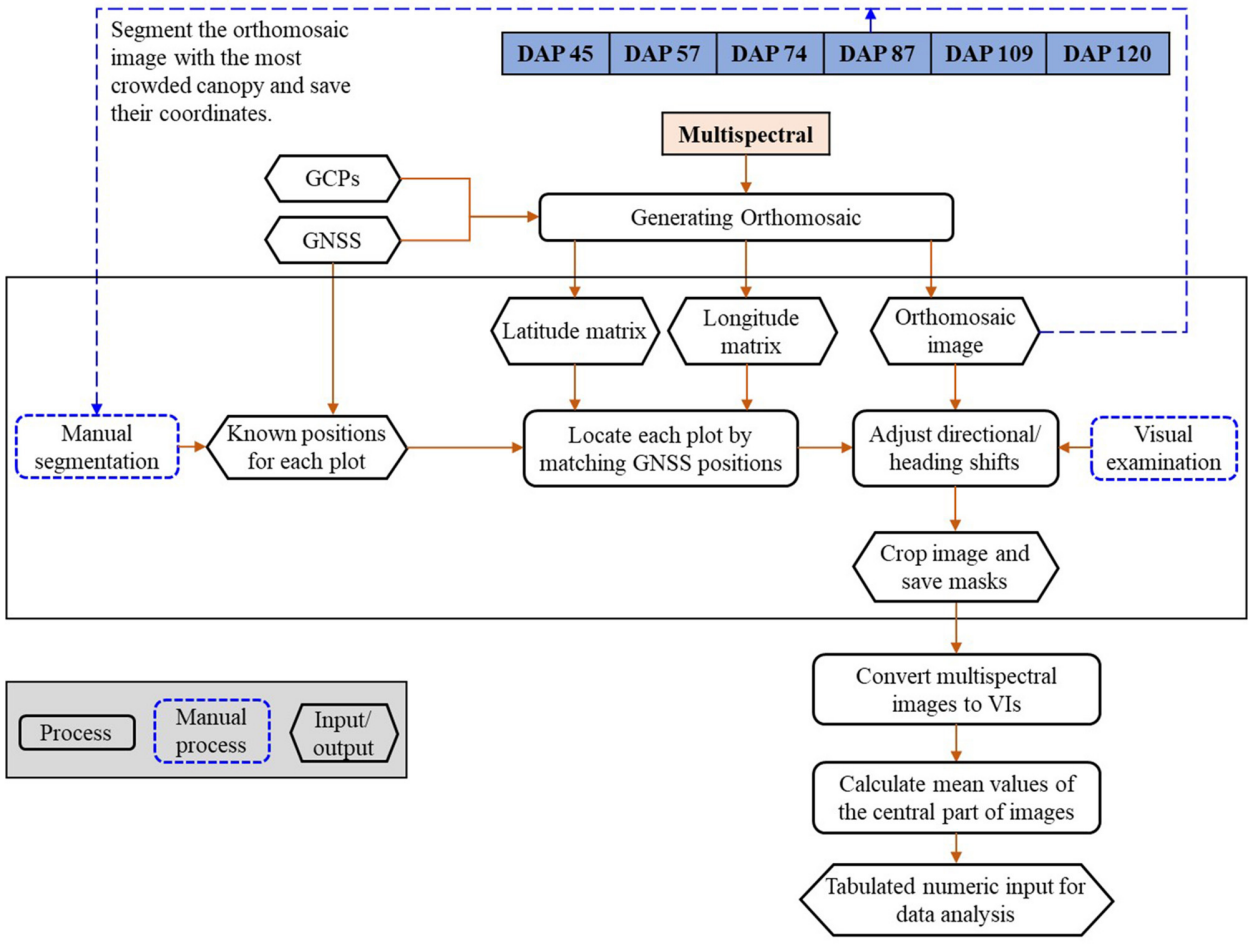
The image processing pipeline.

The images were processed using a pipeline shown in Figure 2, which includes (1) generating orthomosaic images; (2) plot separation; and (3) feature calculation, as shown in Figure 2. The multispectral images were processed using Pix4D Mapper (Pix4D, Lausanne, Switzerland) to generate orthomosaic images and digital elevation models (DEMs) by importing all geo-referenced five-band images and the CRPs for reflectance calibration (Zhou et al., 2019). The generated orthomosaic images and DEMs were then processed using the Mapping Toolbox and Image Processing Toolbox of MATLAB (Version 2019a, The MathWorks, Natick, MA, USA).

Geo-references of the orthomosaic images were used to efficiently separate individual plots from the time-series images. GNSS positions (i.e., latitude and longitude) of the exported orthomosaic images were extracted from the images using the “*pixcenters”* function with a “*makegrid”* option in the Mapping toolbox in MATLAB. The projected geographic coordinate system of the multispectral images was converted into the World Geodetic System 1984 (WGS84) using the function “*projinv” with* required information read from the orthomosaic images. Two matrices were finally returned and each of them had the same dimension as the orthomosaic image from which it was extracted. One of the matrices includes latitude values, while the other includes longitude values.

Individual plots were manually selected from the orthomosaic image built with the images taken when soybean plants had full canopy cover (e.g., DAP 87). A rectangular mask was created to cover the full canopy of each plot and the associated latitude and longitude matrices of the four corners were recorded. The geo-reference information of each plot was used to identify the location of individual plots in the orthomosaic images of other days (e.g., DAP 57). Directional and heading shifts might occur due to the GNSS and stitching errors. The shifts were identified visually and adjusted manually by moving the masks to the right positions. The masks were then applied to the orthomosaic images of DAP 57 to separate the individual plots. The center part of each plot was cropped by removing the image edge (a quarter of image width on each width side and a quarter of image length on each length side). About 38 image features were calculated from the five-band images using the formula summarized by Henrich et al. (2009) and Agapiou et al. (2012). The formula and a brief description of the image features are shown in Supplementary Table 1 and the full descriptions can be found from the Index DataBase (https://www.indexdatabase.de/).

### Model Training and Validation

Soybean breeding lines in the PT and PYT were randomly split into training (80%, i.e., 9,178 and 1,418 lines for the PT and PYT) and testing (20%, i.e., 2,295 and 790 lines for the PT and PYT) datasets to train and validate the variety selection model. The least absolute shrinkage and selection operator (LASSO) model was used in this study for the binary classification problem (James et al., 2013). The LASSO performs both variable selection and regularization to enhance model accuracy and interpretability by adding a penalty term (the L1 norm ||β||_1_) to the least square linear regression (Equation 1).


(1) $$RSS+{\left\|\beta \right\|}_{1}=\;\sum (y_{i}-\beta_{0}-\sum_{j=1}^{p}\beta_{j}x_{ij}{)}^{2}+\text{λ}\sum_{j=1}^{p}\left|\beta_{j}\right|\;$$

where *y*_*i*_ is the response of the *i*th observation, β_0_ is the intercept of the LASSO model, β_*j*_ is the LASSO coefficient of the *j*th predictor, *j* = 1,2,…, *p* and *x*_*ij*_ is the value of the *j*th predictor of the *i*th observation. λ is a tuning parameter of the shrinkage penalty. When λ = 0, the penalty term has no effect, and the LASSO model has little flexibility and will produce the least squares estimates (high variance but no bias). However, as λ approaches ∞, the impact of the shrinkage penalty grows, and the LASSO coefficient estimates of predictors hardly contributing to the model will approach zero. As λ increases, the flexibility of the ridge regression fit decreases, leading to decreased variance but increased bias. The LASSO model improves model accuracy by balancing the bias-variance trade-off to reach the minimum test error (a function of variance plus squared bias). The Lasso model could eliminate potential multicollinearity in the dataset by shrinking the regression coefficients for the highly correlated ones.

Two LASSO models were trained separately for the PT and PYT. The model was built using the “*cv.glmnet*” function in the “*glmnet*” R package by specifying the model type argument *alpha* = 1, *standardize* = “*TRUE*,” and response *family* = “*Gaussian”* with other default arguments. The best shrinkage parameter λ was returned by the “*cv.glmnet*” function (with 5-fold cross-validation on the training set) of choosing the one with the lowest cross-validation error. During the training stage, the LASSO model took 38 image features as predictors and the two classes (0 and 1 in numeric values) as responses and then outputted the probability (0.0–1.0) of a sample being classified to either of the two classes. Those with probabilities higher than the averaged probability were classified as 1 and the rest were as 0. The code for the LASSO model and the imagery datasets can be found at: https://github.com/Heyphil/Soybean-variety-selection.git.

**Figure 3:**
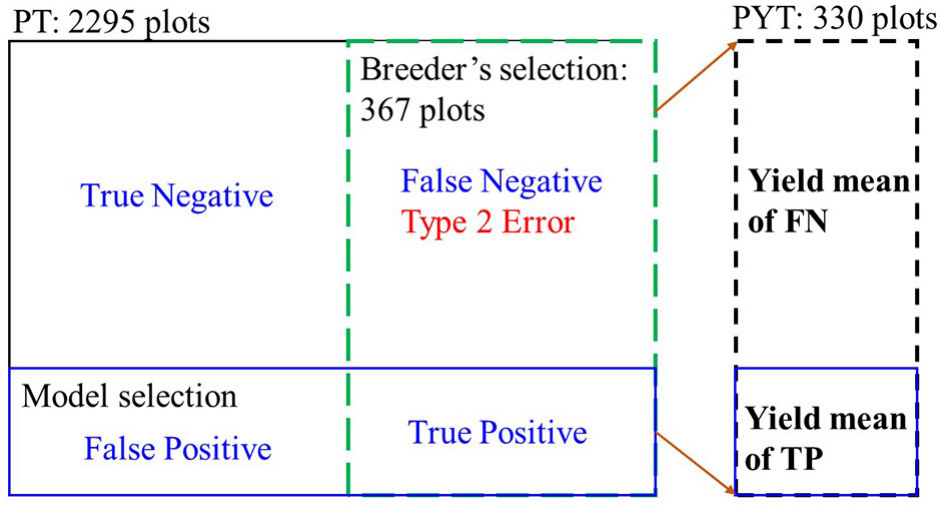
Illustration of two metrics for evaluating model performance in selecting superior soybean breeding lines. The testing sets (20%) included 2,295 and 790 breeding lines for the PT and PYT.

As our goal in this study was to assist breedzers to identify elite lines that have higher yield potential in the following years from their selected group, we focused on avoiding misclassification of the selected lines to the non-selected group, i.e., minimizing the false negative (FN) rates, which can be quantified by the classification recall (Eq. 2) of the testing sets. As shown in Figure 3, the true positive (TP) class represents the overlapping between the model selections and the selection of breeder, while the FN class contains those who were not selected by the model but selected by the breeder. In this study, the FN class needs to be avoided, i.e., the minimum type 2 error. Recall measures the proportion of the TP class out of all the breeder selections. For imbalanced datasets like this case (only around 10% of one class while 90% of the other), F1-score of the classification model was calculated (Eq. 3) to take both the FN rates (recall) and false positive rates (precision) and into account at equal weights (Cao et al., 2020). It returns values between 0.0 (the worst model performance) and 1.0 (the best model performance).

In breeding programs, it is always preferred that the selected genotypes have an increased yield from one generation to the next (Moreira et al., 2019). To evaluate the model performance, yield means in the following YTs of the TP class (the model selections) were compared to the yield means of the FN class (the model non-selected group but selected by breeders phenotypically).


(2) $$\begin{array}{r} Recall=\frac{TP}{TP+FN} \end{array}$$

(3) $$\begin{array}{r} \mathrm{F1-score}=\frac{2\;\times TP}{2\times TP+FP+FN} \end{array}$$

where, TP is the true positive class, FP is the false positive class, and FN is the false negative class.

### Data Analysis

Data analyses were conducted using *R* language (Team, 2021) in RStudio (Ver. 1.3.1093, RStudio, Boston, MA, USA). A one-way ANOVA with an honest significant difference Tukey test was conducted to evaluate the differences in yield, plant height, and lodging between the selected and non-selected soybean breeding lines. Principal component analysis (PCA) was performed to estimate the relationship among the manually measured agronomic traits and model the selection criterion of a breeder. PCA is an unsupervised approach allowing to summarize a dataset with principal components (PCs). Each PC is a normalized linear combination of features that collectively explain most of the variability in the original set (James et al., 2013). The PCA was performed using the “*prcomp*” function to visualize the feature contributions to PC models and their relationship.

## Results

### Efficacy of the Manual Selection

**Figure 4:**
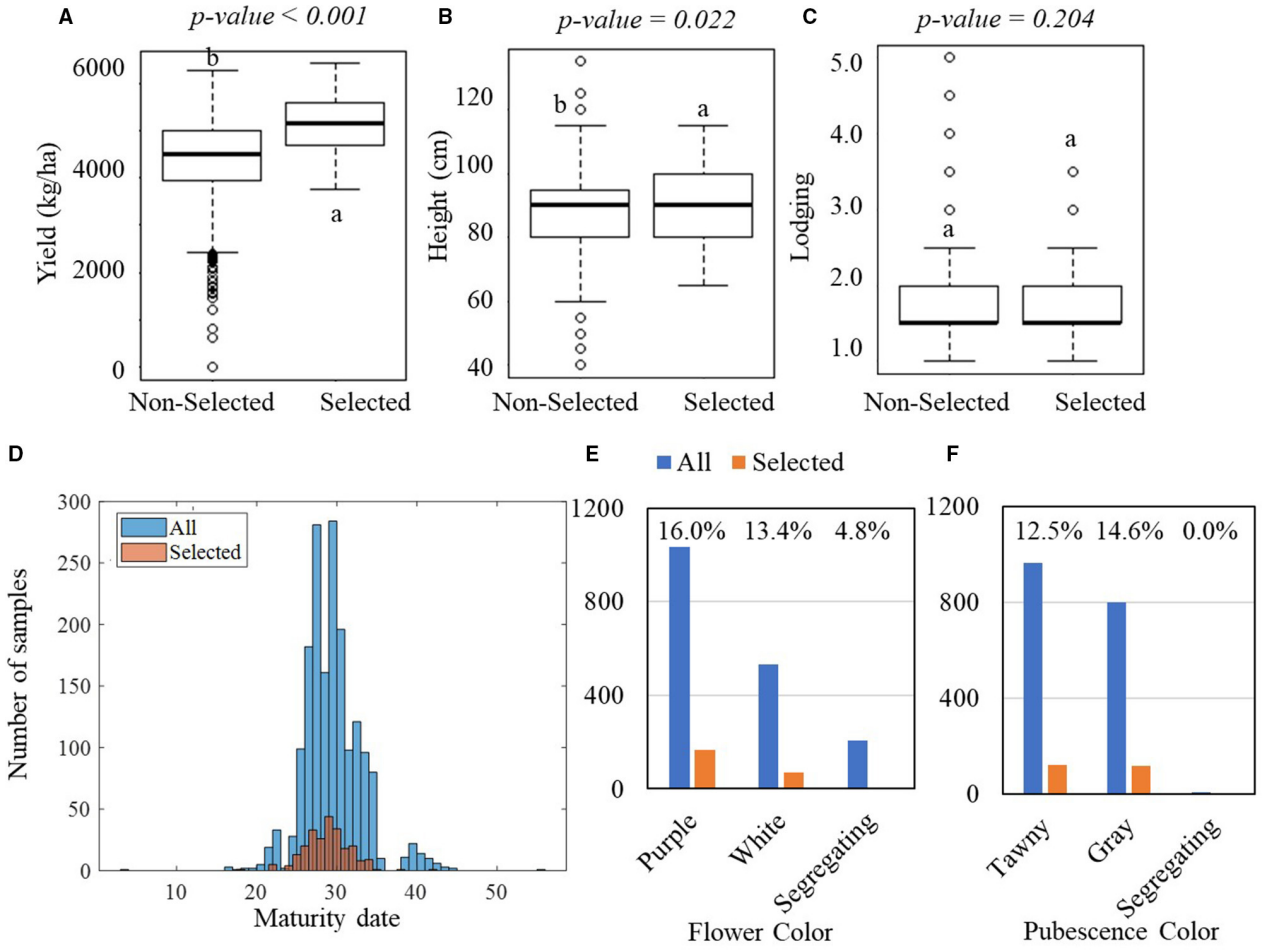
Comparisons of manually measured agronomic traits between the selected and non-selected groups in the PYT. In the PYT, 238 breeding lines were selected, while 1,535 breeding lines were non-selected. **(A–C)** Boxplots of yield, plant height, and lodging for the selected and non-selected groups. The lower-case letters above bars indicate the significant difference among these means at the above significance levels (*p-values*). **(D)** Histogram of the maturity dates. **(E,F)** Bar plots of the flower and pubescence colors. The blue bars represent all plots, while the orange bars represent the selected plots.

**Figure 5:**
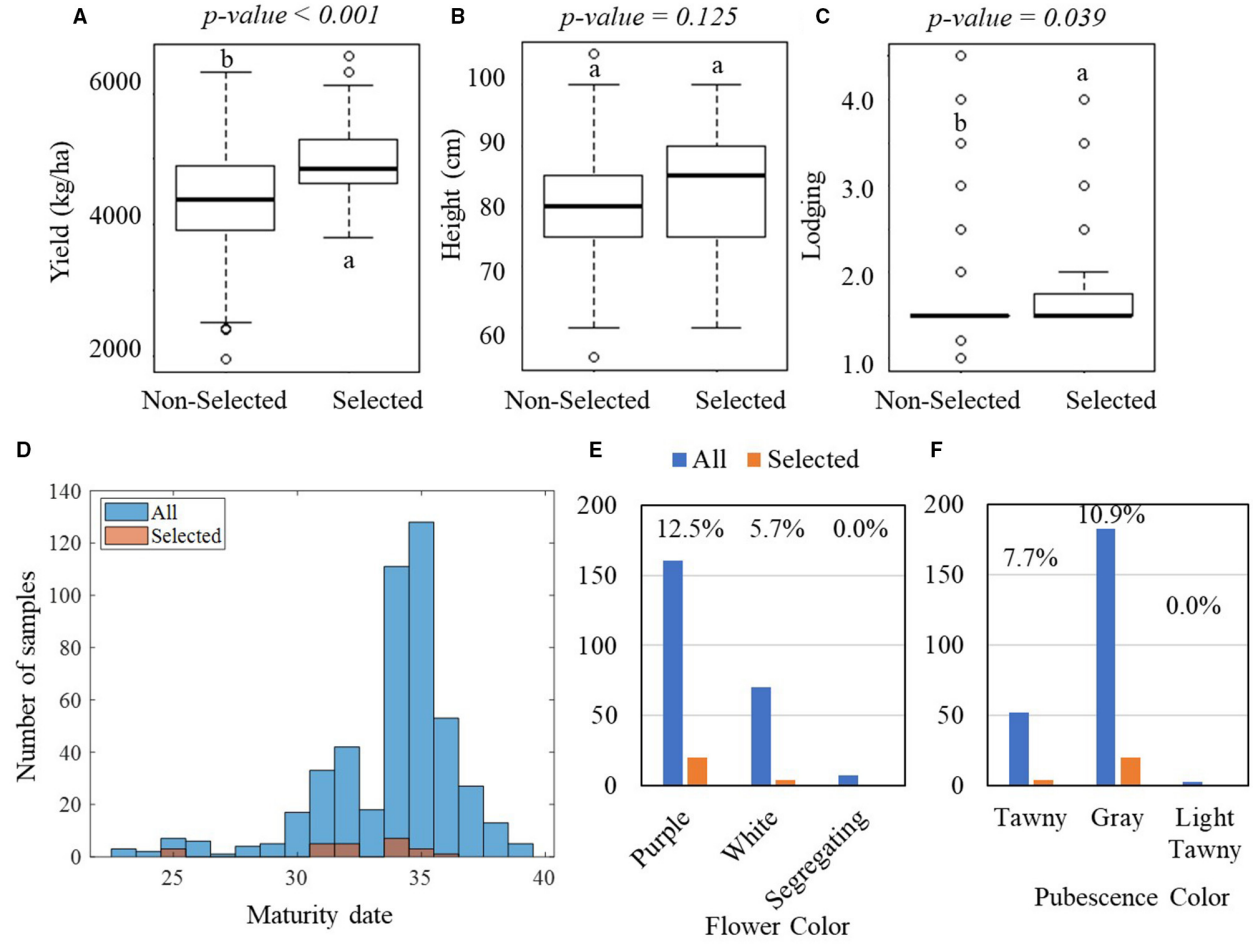
Comparisons of manually measured agronomic traits between the selected and non-selected groups in the AYT. In the AYT, 44 breeding lines were selected, while 194 breeding lines were non-selected. **(A–C)** Boxplots of the comparisons in yield, plant height, and lodging, respectively. The lower-case letters above bars indicate the significant difference among these means at the above significance levels (*p-values*). **(D)** Histogram of the maturity dates. **(E,F)** Bar plots of the flower and pubescence colors. For **(E,F)**, the blue bars represent all breeding lines, while the orange bars represent the selected breeding lines.

Figure 4 shows the result of one-way ANOVA tests on the manually measured agronomic traits that were collected in the YTs. In the PYT, significant differences were observed in yield between the selected and non-selected groups, with the selected group yielding on average 5,120 kg·ha^−1^ with an SD of 584 kg·ha^−1^ and the non-selected group yielding 4,423 kg·ha^−1^ with an SD of 778 kg·ha^−1^ (Figure 4A). The yield difference was observed consistently in the AYT, with the selected group averagely producing 4,965 kg·ha^−1^ yield with an SD of 622 kg·ha^−1^ and the non-selected group 4,384 kg·ha^−1^ with an SD of 770 kg·ha^−1^ (Figure 5A). In the PYT, plant height had a dynamic range from 45.0 to 125.0 cm and the selected group (89.6 cm, SD = 10.3 cm) was significantly taller than the non-selected one (87.9 cm, SD = 11.2 cm), while in the AYT there was no difference in plant height (55.0–105.0 cm) between the two groups (Figure 5B). It is noted that the selected group had higher lodging readings than the non-selected group, which was possibly caused by the fewer variations in lodging, as most of the breeding lines in the AYT (Figure 5C) had the lodging readings within the range of 1–2, except for a few breeding lines over 3. For the maturity date, the selected groups were mostly located between 25–37 and 30–37 days after September 1 (Figure 5D). From Figures 4E,F, 5E,F, flower and pubescence colors were not the decisive factors as specific percentages of selection were made from each of the colors. However, color segregating was an indicator of the non-selection groups. The result indicates that the breeders made selection primarily on yield performance and eliminating those with undesired secondary traits, such as early or late MG and high lodging rate.

### Modeling Breeder's Selection Criterion

**Figure 6:**
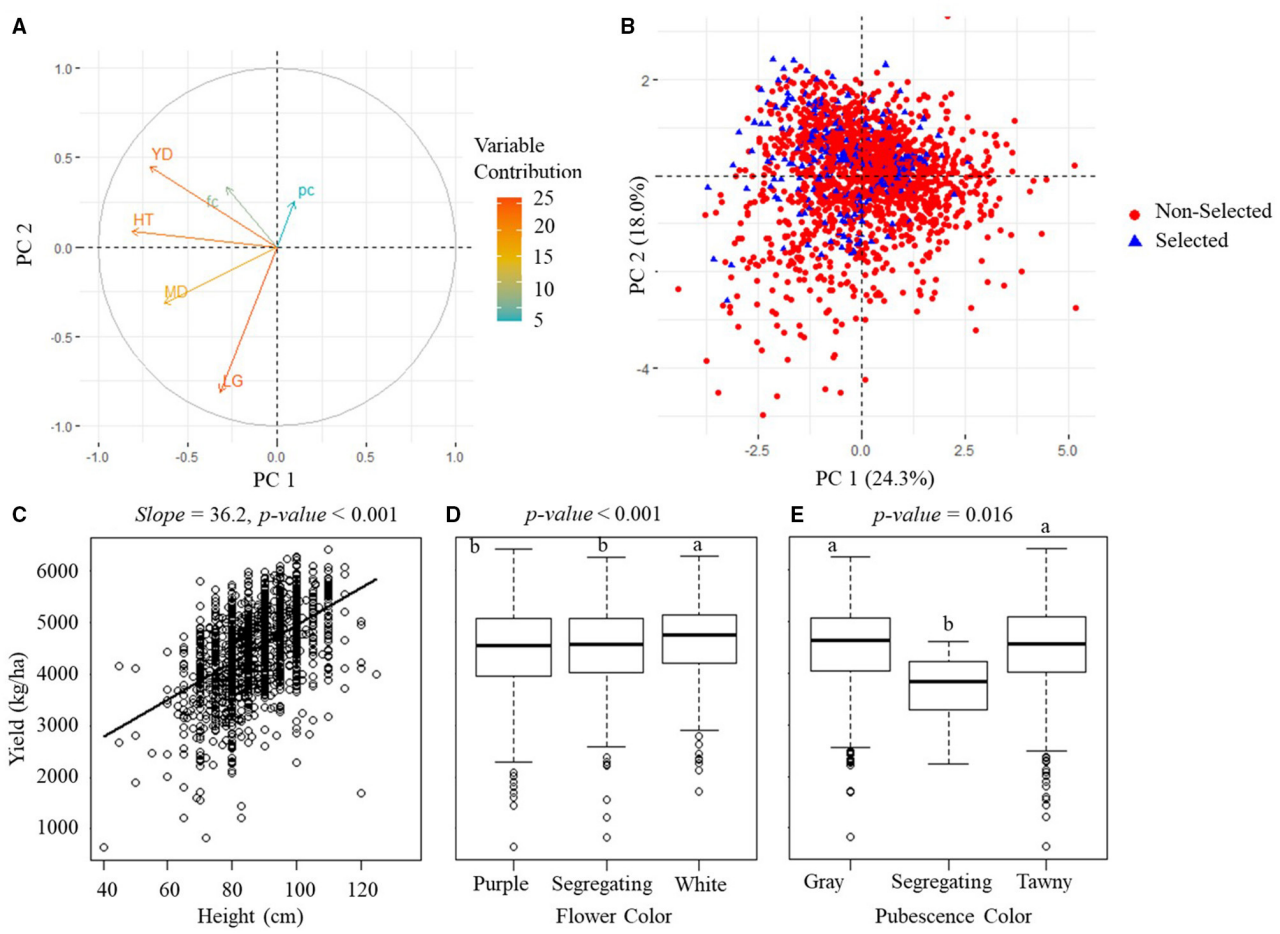
Manually measured agronomic traits in PYT. **(A)** The visualization plots of the variables' contributions and directions in the plane of the first two PCs. YD, yield; HT, plant height; MD, maturity date; LG, lodging; fc, flower color; pc, pubescence color. **(B)** The projection of sample variables on the first two PCs. **(C)** The scatterplot of yield vs. plant height. **(D,E)** The boxplots of the yield of three groups of flower color and three groups of pubescence color. The *p-value* above **(C–E)** indicates the significance level of estimation.

**Figure 7:**
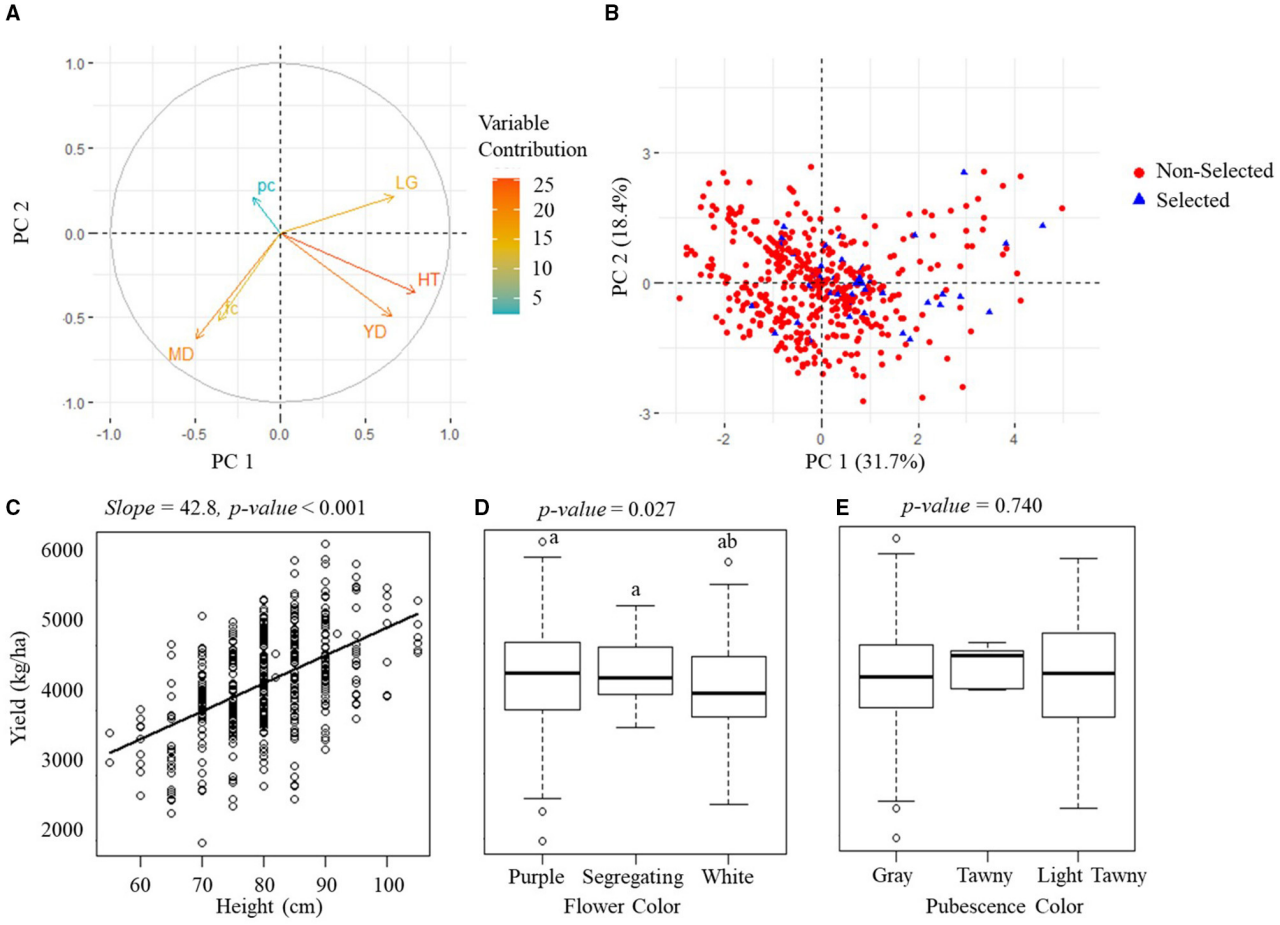
Manually measured agronomic traits in AYT. **(A)** The visualization plots of the variables' contributions and directions in the plane of the first two PCs. YD, yield; HT, plant height; MD, maturity date; LG, lodging; fc, flower color; pc, pubescence color. **(B)** The projection of sample variables on the first two PCs. **(C)** The scatterplot of yield vs. plant height. **(D,E)** are boxplots of the yield of three groups of flower color and three groups of pubescence color. The *p-value* above **(C–E)** indicates the significance level of estimation.

The PCA analytical results on the manually measured agronomic traits for the PYT and AYT are shown in Figures 6A, 7A. The PCA revealed three PCs with eigenvalues > 1 capturing 65.3% of the total variation for the PYT and two PCs capturing 51.1% for the AYT. In both YTs, the first two PCs were mainly influenced by yield, plant height, maturity date, and lodging. Yield and plant height had the highest contributions to PC1 for both trials and pointed in a similar direction. It was also noted from Figures 6C, 7C that plant height was positively correlated with yield. The lodging and maturity date had the second important contribution to the PCA models. Among breeding lines with high rankings in yield, those with severe lodging and not in the targeted MGs will not be selected.

Flower and pubescence color had little contribution to the selection. In PYT, soybean breeding lines with the white flower color had a higher yield than those with purple and segregating, while those with the pure pubescence colors had a higher yield than segregating lines. In AYT, however, no significant difference was found among different colors, which may be due to the fewer variations in the AYT breeding lines. The relationship between yield and other agronomic traits shown in Figures 6, 7 generally confirms the variable contributions found in the first two PCs in the PYT and AYT.

Figures 6B, 7B show the scatterplots of sample distribution based on PCA regression scores, with different colors for the selected and non-selected groups. The two groups mainly overlapped with each other in the first two PCs for both trials. However, it can be seen that the selected group tended to follow the trend of high yield (i.e., negatively directing to the axis of PC1 for the PYT while positively directing to PC2 for the AYT). There was no clear difference between the two groups in the axis of PC2 that were dominated by maturity date and lodging.

### Differences in Image Features Between Selected and Non-selected Breeding Lines

**Figure 8:**
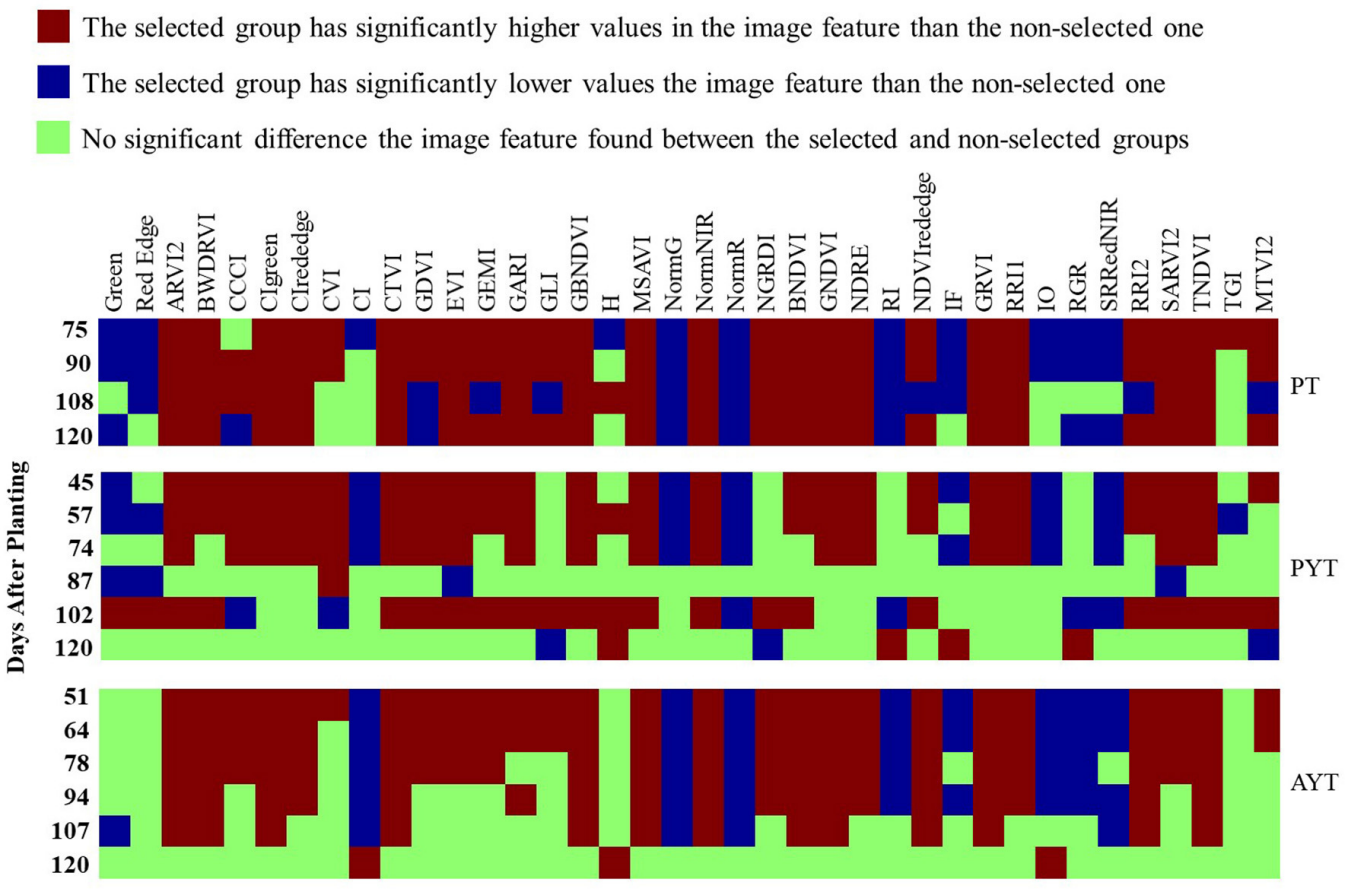
The significance map of the differences in image features between the selected and non-selected groups. Each square represents if there is a significant difference in an image feature collected at single times (*p* < 0.05) between the two groups.

Figure 8 shows the significance of the differences in 38 image features collected multiple times in the PT, PYT, and AYT. Each square indicates the type of difference (significantly higher, lower, or no difference) in the mean of an image feature (all listed above) between the selected and non-selected groups. The vertical axes were the DAPs when data were collected in each trial, and trials were indicated on the right side of the graph. In the PT, there were 37, 35, 31, and 31 image features significantly different between the two groups, collected on the DAPs of 75, 90, 108, and 120, respectively. There were 31, 32, 24, 4, 28, and 7 image features in the PYT and 34, 33, 28, 26, 18, and 3 image features in the AYT that showed significant differences from the first to the last data collection. Among the 38 image features, 17 had consistently significant patterns (i.e., the selected group consistently higher or lower than the non-selected group) for the three trials. In general, there was a less significant difference in advanced trials due to the less variation among selected breeding lines than those in previous trials.

The PT maintained a relatively consistent number of image features with significant differences from the mid- (DAP 78) to late- (DAP 120) growth stages. While in the PYT and AYT, most of the image features were significant before the 90 DAPs; however, there were fewer distinguishable features when the plants started entering the maturity stages (R6). Therefore, the image features at early- to mid-stages (R3–R5) show more potential to select superior breeding lines, especially YTs. It is expected that model-based selection may shorten the breeding cycle by eliminating less-potential breeding lines at early stages.

### Performance of the Model-Based Breeding Line Selection

**Figure 9:**
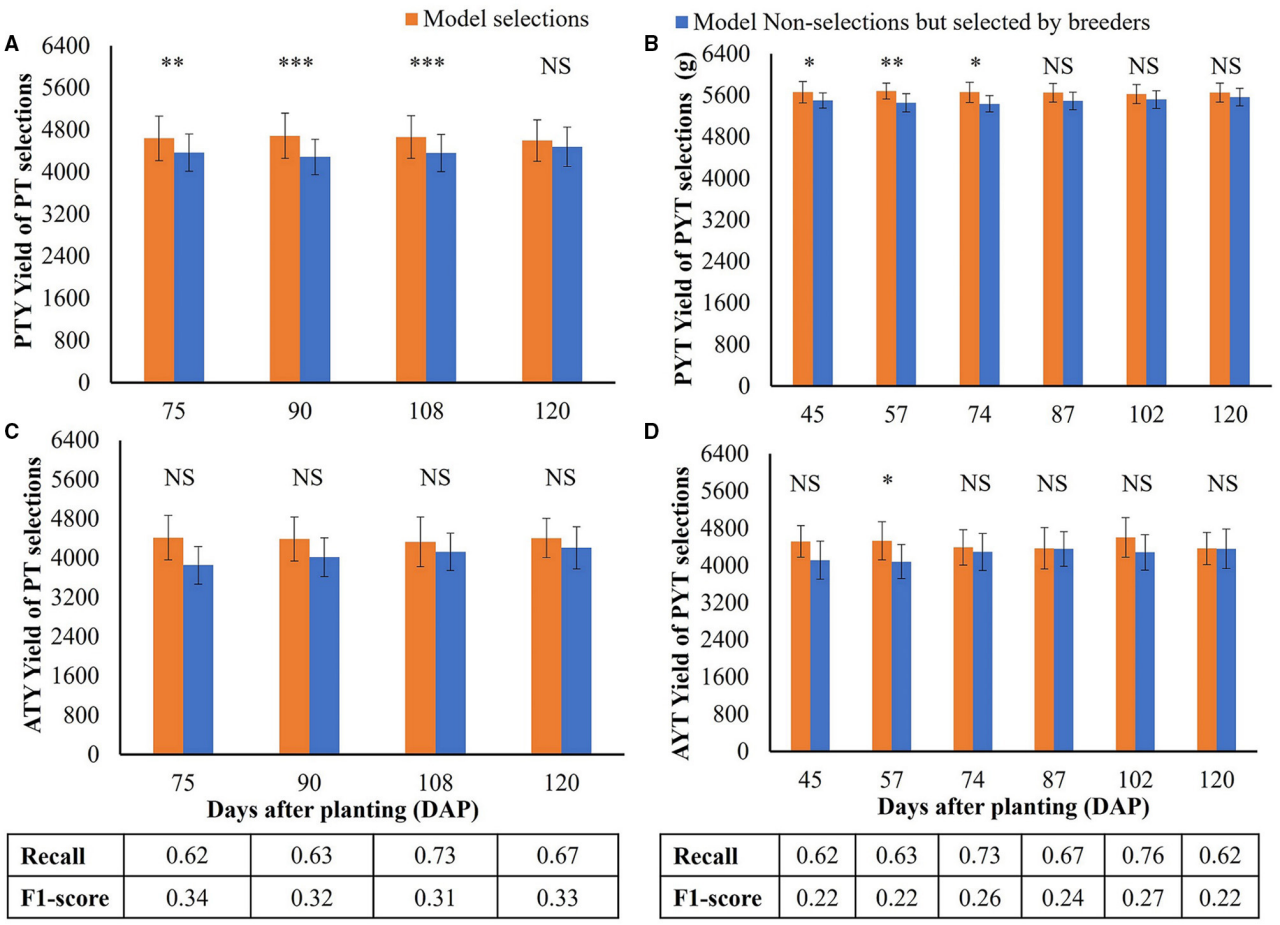
Model performance on selecting superior soybean breeding lines from the PT and PYT. **(A,C)** Comparison of the PYT yield means. Yield unit: kg·ha^−1^. **(B,D)** Comparison of the AYT yield means. **(A,B)** are for the PT selections, while **(C,D)** are for the PYT selections. Orange and blue bars represent the model selections (the TP class) and model non-selections but selected by breeders (the FN class), respectively. Error bars show the standard deviations. *, **, and *** indicate the significance levels of *p* = 0.05, 0.01, and 0.001 for testing the hypothesis that there is no difference in yield means between model selections and non-selections. NS represents no significance. The numbers below the bar plots are the classification recall and F1-score for the model on each day.

The yield performance of the selected breeding lines based on the LASSO model (model-based selection) for the PT and PYT is shown in Figure 9. As the LASSO model has the advantage of handling multicollinearity effects among model input, all the 38 image features were used in the model without preliminary feature selection. The numbers below the bar plots show the classification recalls representing the proportions of the selection of breeder that were also selected by the models and F1-scores representing the overall model performance, based on imagery data collected at different times. For both PT and PYT, the recalls were above 0.60 for the testing sets, indicating that the models were able to identify over 60% of the selections of a breeder. The highest recalls were 0.71 (90 DAPs) and 0.76 (102 DAPs) for the PT and PYT, respectively. In all the breeder's selected breeding lines in PT, the breeding lines also selected by the LASSO model had a significantly higher yield in PYT than the non-selected lines, when the model used image features collected at 75, 90, and 108 DAPs (Figure 9A). No significant difference in yield was observed when using the image features on the 120 DAPs. However, Figure 9B shows that there was no significant difference in yield between the two groups in the 2020 AYT. Figures 9C,D show the performance of model-selected breeding lines based on imagery data collected in PYT. Figure 9C shows that in the selection of breeder of the PYT breeding lines, the yield of breeding lines selected by models was significantly higher than those not selected by models when using image features at earlier stages (45, 57, and 74 DAPs). However, there was no significant difference in yield between breeding lines selected and non-selected by models in the AYT, except at the 57 DAPs.

Compared the breeding lines selected by models with the breeding lines selected by breeders in PT, yield in the PYT of the model selection (90 DAPs) were up to 4% (201 kg·ha^−1^) higher than the selection of breeder and 2% (103 kg·ha^−1^) higher in the AYT. In addition, the yield of the model selection (57 DAPs) was up to 2% (112 kg·ha^−1^) higher than the selection of breeder in the PYT and 5% (223 kg·ha^−1^) higher in the AYT. It is noted from Figure 9C that the highest recall (0.76) and the highest F1-score (0.27) in the PYT selections were obtained using image features on the 102 DAPs, but the PYT yield of the model selection was not significantly higher than the remaining breeding lines in the selection of a breeder. A similar situation was observed for PT selections on the 120 DAPs. Therefore, the high classification recall or F1-score did not necessarily lead to higher yield performance in the following trials.

The model selections based on image features collected before the maturity stage (i.e., 75, 90, and 108 DAPs in Figure 9A) had significantly higher PYT yield than the non-selections. Although there was no significant difference in the AYT yield in the corresponding DAP due to higher variations, the mean yield of breeding lines selected by the model on the 75 and 90 DAPs was higher than that of the selection of a breeder. Similarly, the significance was observed in the PYT model selections at even earlier stages before the 87 DAPs (the beginning of seed fill). Thus, image-based crop traits collected before maturity tended to perform better in selecting higher-yielding progeny breeding lines and, the appropriate time point (at the R3–R5 stages) for making selections for YTs should be even earlier due to the less variation in these trials.

## Discussion

Grain yield is the only trait that had significant differences between selected and non-selected breeding lines (Figures 4A, 5A) in both PYT and AYT and yield consistently and highly contributed (Figures 6A, 7A) to explain the variations in collected agronomic traits. Yield estimation using UAV-based remote sensing technologies has been a hot topic in the area of HTP, and yield has shown high correlations with image features (Herrmann et al., 2019; Moreira et al., 2019; Maimaitijiang et al., 2020). In one of our previous studies (Zhou et al., 2021b), we found that yield was highly correlated with vegetation indices that were also shown to be significantly distinguishable for selecting superior soybean breeding lines in all three trials (Figure 8). The results imply that the reliability of using these features to make selections in scenarios where yield collection is unfeasible, i.e., the PT.

Similar to yield, plant height is another agronomic trait that highly contributed to explaining the variations in both YTs, which might be due to the significantly positive correlation between yield and plant height (Figures 6C, 7C). However, plant height is not necessarily correlated with yield in soybean breeding experiments as soybean genotypes with different growth habits or MGs developed different strategies for nutrient partitioning and sink/source distribution of energy (Purcell et al., 2014). It should be noted that the soybean materials in this study were all indeterminate (IND) varieties that continue their vegetative growth and produce nodes on the main stem until the beginning of seed fill (R5) (Purcell et al., 2014). Compared to those with a determinate (DET) growth habit, i.e., stop vegetative growth and producing nodes before the flowering stages, IND soybeans usually are tall and have a high yield. However, they are more susceptible to lodging. It was reported by Wilcox and Sediyama (1981) that seed yield would increase 350 kg·ha^−1^ and lodging score would increase 0.008 (on a score scale of 1–5) when plant height increased every 10 cm for DET breeding lines. On the other hand, seed yield increased 112 kg·ha^−1^ and lodging score increased 0.3 for every 10 cm increase in plant height for IND breeding lines. Therefore, plant lodging is non-negligible in soybean breeding, and those with severe lodging have to be avoided, especially among the IND varieties because of its tall plant height due to elongation of the main stem after flowering (Kato et al., 2015).

Severe lodging was not observed in this study with only six and seven breeding lines having lodging scores >3 in the PYT and AYT, respectively. Moreover, there was not a consistently incremental or decremental relationship shown between lodging and yield (Figures 6D, 7D). It has been shown that lodging from R3 (beginning pod filling) to R5 (beginning seed filling) have the greatest impact on yield, while lodging before R2 (full flowering) and after R6 (full seed) does not significantly impact yield, but lodging at the maturity stage can have negative impacts on harvest ability (Koester et al., 2014). As the lodging scores were taken when soybeans reached their R8 stages, they did not necessarily reflect the adverse effects on the yield of potential lodging in the middle of the growing seasons.

Figure 8 gives a general idea of how differentiable the image features were between selected and non-selected breeding lines over the growing seasons and the consistent differences in multiple years and locations. Significant differences were observed in many image features between the selected and non-selected breeding lines in all three trials. The PT had consistent differences from the mid- to late-growth stages. On the contrary, the PYT and AYT breeding lines had fewer distinguishable features after around 80 DAPs. In soybean breeding, the selection on progeny row is usually based on the breeder's visual examinations. The breeding lines with a similar appearance to the surrounding checks would be preferable and have desired performance regarding yield potential and maturity date. Therefore, the selected progeny rows are expected to have low variations compared to all the materials planted in the PT.

Compared to the selections of a breeder, the model selections based on image features collected at early growth stages had better performance in selecting superior breeding lines. The model selections had a significantly higher yield in the next year. Due to higher variations in the PT materials, superior progeny rows were easier to be identified with image features collected through early reproductive stages (75–108 DAPs). With the model selection, nearly 30% of soybean breeding lines could be cut off in the following YTs, consequently saving 30% of land usage, human labor, and other resources. With the aid of efficient data collection and selection, human error could be reduced from the traditional breeding pipeline, and the limitation on the number of progeny rows could be boosted. Hence the genetic gain could be increased by a higher selection intensity benefited from higher population size.

## Conclusion

This study evaluated the performance of a UAV-based HTP system in the selection of superior soybean breeding lines for a breeding program. A total of 11,473 progeny rows were planted in 2018 (PT), and 1,773 among them were selected for the PYT in 2019 and 238 were then selected for the AYT in 2020. Six agronomic traits, including yield, plant height, maturity data, flower and pubescence color, and lodging, were measured for soybeans in PYT and AYT. Unmanned Aerial Vehicle-based images were collected every 2 weeks over the growing seasons, and a group of image features was extracted from five-band multispectral images for each trial. Research results show that yield is the primary trait for selecting superior soybean lines by breeders as there were significant differences in yield between the selected and non-selected groups for both PYTs and AYTs. It was also found that progeny rows had the most variation among the trials, and the images collected at earlier stages (before R5) explained more variation than those at later stages, consistently for the PT, PYT, and AYT. The LASSO model for selecting soybean breeding lines with image features correctly identified 71% and 76% of the selection of breeder for the PT and PYT. The PYT yield in PT model selections before 120 DAPs and the PYT model selection before 87 DAPs was significantly higher (*p* < 0.05) than the model non-selection but selected by breeders. The model selections in PT and PYT had, respectively, 4 and 5% higher yield in the following year's trial, comparing the selection of a breeder. It could be concluded that the proposed model is promising in making selections on soybean breeding trials.

## Data Availability Statement

The raw data supporting the conclusions of this article will be made available by the authors, without undue reservation.

## Author Contributions

JiaZ and JinZ conceived the idea and developed research questions. JinZ collected and analyzed the data and prepared the original draft. EB managed the field experiment, collected the field data, and reviewed and edited the manuscript. CV reviewed and edited the manuscript. DY managed the field experiment and collected the field data. JiaZ supervised the data collection, analysis, and interpretation and reviewed and edited the manuscript. AS and PC supervised the data source and field experiment, and reviewed and edited the manuscript. All authors contributed to the article and approved the submitted version.

## Funding

This study was supported by the Missouri Soybean Merchandising Council.

## Conflict of Interest

The authors declare that the research was conducted in the absence of any commercial or financial relationships that could be construed as a potential conflict of interest.

## Publisher's Note

All claims expressed in this article are solely those of the authors and do not necessarily represent those of their affiliated organizations, or those of the publisher, the editors and the reviewers. Any product that may be evaluated in this article, or claim that may be made by its manufacturer, is not guaranteed or endorsed by the publisher.

## References

[B1] Agapiou A. Hadjimitsis D. G. Alexakis D. D. (2012). Evaluation of broadband and narrowband vegetation indices for the identification of archaeological crop marks. Remote Sensing 4:3892. 10.3390/rs4123892

[B2] Ahmar S. Gill R. A. Jung K.-H. Faheem A. Qasim M. U. Mubeen M.. (2020). Conventional and molecular techniques from simple breeding to speed breeding in crop plants: recent advances and future outlook. Int. J. Mol. Sci. 21:2590. 10.3390/ijms21072590 32276445 PMC7177917

[B3] Araus J. L. Cairns J. E. (2014). Field high-throughput phenotyping: the new crop breeding frontier. Trends Plant Sci. 19, 52–61. 10.1016/j.tplants.2013.09.008 24139902

[B4] Araus J. L. Kefauver S. C. Zaman-Allah M. Olsen M. S. Cairns J. E. (2018). Translating high-throughput phenotyping into genetic gain. Trends Plant Sci. 23, 451–466. 10.1016/j.tplants.2018.02.001 29555431 PMC5931794

[B5] Arruda M. P. Lipka A. E. Brown P. J. Krill A. M. Thurber C. Brown-Guedira G.. (2016). Comparing genomic selection and marker-assisted selection for Fusarium head blight resistance in wheat (*Triticum aestivum* L.). Mol. Breed. 36:84. 10.1007/s11032-016-0508-5

[B6] Bai G. Jenkins S. Yuan W. Graef G. L. Ge Y. (2018). Field-based scoring of soybean iron deficiency chlorosis using RGB imaging and statistical learning. Front. Plant Sci. 9:1002. 10.3389/fpls.2018.01002 30050552 PMC6050400

[B7] Beche E. Gillman J. D. Song Q. Nelson R. Beissinger T. Decker J.. (2020). Nested association mapping of important agronomic traits in three interspecific soybean populations. Theoretical Appl. Gene. 133, 1039–1054. 10.1007/s00122-019-03529-4 31974666

[B8] Cao C. Chicco D. Hoffman M. M. (2020). >The MCC-F1 curve: a performance evaluation technique for binary classification. arXiv preprint arXiv:2006.11278. Available online at: https://arxiv.org/abs/2006.11278.

[B9] Emlid (2021). Reach RS/RS+ docs: Placing the Base. Available online at: https://docs.emlid.com/reachrs/common/tutorials/placing-the-base/ (accessed December 15, 2021).

[B10] Fehr W. R. (1991). Principles of Cultivar Development: Theory and Technique. New York, NY: Macmillian.

[B11] Henrich V. Götze E. Jung A. Sandow C. Thürkow D. Gläßer C. (2009). Development of an Online Indices-Database: Motivation, Concept and Implementation. EARSeL proceedings, EARSeL, Tel Aviv.34033582

[B12] Herrmann I. Bdolach E. Montekyo Y. Rachmilevitch S. Townsend P. A. Karnieli A. (2019). Assessment of maize yield and phenology by drone-mounted superspectral camera. Precision Agriculture 21, 51–76. 10.1007/s11119-019-09659-5

[B13] James G. Witten D. Hastie T. Tibshirani R. (2013). An Introduction to Statistical Learning. New York, NY: Springer.

[B14] Kato S. Fujii K. Yumoto S. Ishimoto M. Shiraiwa T. Sayama T.. (2015). Seed yield and its components of indeterminate and determinate lines in recombinant inbred lines of soybean. Breed. Sci. 65, 154–160. 10.1270/jsbbs.65.154 26069445 PMC4430510

[B15] Khaki S. Pham H. Han Y. Kuhl A. Kent W. Wang L. J. S. (2020). Convolutional neural networks for image-based corn kernel detection and counting. Sensors 20:2721. 10.3390/s20092721 32397598 PMC7249160

[B16] Koester R. P. Skoneczka J. A. Cary T. R. Diers B. W. Ainsworth E. A. (2014). Historical gains in soybean (*Glycine max* Merr.) seed yield are driven by linear increases in light interception, energy conversion, and partitioning efficiencies. J. Exp. Bot. 65, 3311–3321. 10.1093/jxb/eru187 24790116 PMC4071847

[B17] Maimaitijiang M. Sagan V. Sidike P. Hartling S. Esposito F. Fritschi F. B. (2020). Soybean yield prediction from UAV using multimodal data fusion and deep learning. Remote Sens. Environ. 237:111599. 10.1016/j.rse.2019.111599

[B18] Mammadov J. Aggarwal R. Buyyarapu R. Kumpatla S. (2012). SNP markers and their impact on plant breeding. Int. J. Plant Genomics 2012:11. 10.1155/2012/728398 23316221 PMC3536327

[B19] Moreira F. F. Hearst A. A. Cherkauer K. A. Rainey K. M. (2019). Improving the efficiency of soybean breeding with high-throughput canopy phenotyping. Plant Methods 15:139. 10.1186/s13007-019-0519-4 31827576 PMC6862841

[B20] Orf J. H. Diers B. W. Boerma H. R. (2004). Genetic improvement: Conventional and molecular-based strategies. Soybeans 16, 417–450. 10.2134/agronmonogr16.3ed.c915991677

[B21] Pratama M. T. Kim S. Ozawa S. Ohkawa T. Chona Y. Tsuji H.. (2020). “Deep learning-based object detection for crop monitoring in soybean fields,” in Paper presented at the 2020 International Joint Conference on Neural Networks (IJCNN), (Glasgow: IEEE).

[B22] Purcell L. C. Salmeron M. Ashlock L. (2014). Soybean growth and development. Arkansas Soybean Production Handbook 197, 1–8. Available online at: https://www.uaex.uada.edu/publications/pdf/MP197/chapter2.pdf

[B23] Richards R. (2000). Selectable traits to increase crop photosynthesis and yield of grain crops. J. Experi. Botany 51(suppl_1), 447–458. 10.1093/jexbot/51.suppl_1.447 10938853

[B24] Sleper D. A. Poehlman J. M. (2006). Breeding Field Crops. Oxford: Blackwell publishing.

[B25] Team R. C. (2021). R: A Language and Environment for Statistical Computing. Available online at: https://www.R-project.org/ (accessed December 15, 2021).

[B26] Vieira C. C. Chen P. J. C. B. Biotechnology A. (2021). The numbers game of soybean breeding in the United States. Crop Breed. Appl. Biotechnol, 21. 10.1590/1984-70332021v21Sa23

[B27] Wilcox J. R. Sediyama T. (1981). Interrelationships among height, lodging and yield in determinate and indeterminate soybeans. Euphytica 30, 323–326. 10.1007/BF00033993

[B28] Xu Y. Li P. Zou C. Lu Y. Xie C. Zhang X.. (2017). Enhancing genetic gain in the era of molecular breeding. J. Exp. Bot. 68, 2641–2666. 10.1093/jxb/erx135 28830098

[B29] Zhou J. Mou H. Zhou J. Ali M. L. Ye H. Chen P.. (2021a). Qualification of soybean responses to flooding stress using uav-based imagery and deep learning. Plant Phenomics 2021:9892570. 10.34133/2021/9892570 34286285 PMC8261669

[B30] Zhou J. Yungbluth D. Vong C. N. Scaboo A. Zhou J. (2019). Estimation of the maturity date of soybean breeding lines using UAV-based multispectral imagery. Remote Sensing 11:2075. 10.3390/rs11182075

[B31] Zhou J. Zhou J. Ye H. Ali M. L. Chen P. Nguyen H. T. (2021b). Yield estimation of soybean breeding lines under drought stress using unmanned aerial vehicle-based imagery and convolutional neural network. Biosyst. Eng. 204, 90–103. 10.1016/j.biosystemseng.2021.01.017

[B32] Zhou J. Zhou J. Ye H. Ali M. L. Nguyen H. T. Chen P. (2020). Classification of soybean leaf wilting due to drought stress using UAV-based imagery. Comp. Electr. Agri. 175:105576. 10.1016/j.compag.2020.105576

