# Supplementary material for: Improve Soybean Variety Selection Accuracy Using UAV-Based High-Throughput Phenotyping Technology
Source: Front Plant Sci. 2022 Jan 11;12:768742. doi: 10.3389/fpls.2021.768742 (PMC8786709; doi:10.3389/fpls.2021.768742)
Supplement: Supplementary file 1 [file Table_1.docx]

Supplementary

**Table S1.** A list of 36 vegetation indices (VIs) used in the LASSO models.

| **No.** | **Index Name** | **Descriptions** | **Formula** |
| --- | --- | --- | --- |
| 1 | ARVI2 | Atmospherically Resistant VI 2 | $-0.18+0.17\times\frac{nir-red}{nir+red}$ |
| 2 | BWDRVI | Blue-wide dynamic range VI | $\frac{0.1\times nir-red}{0.1\times nir+red}$ |
| 3 | CCCI | Canopy Chlorophyll Content Index | $\frac{\frac{nir-re}{nir+re}}{NDVI}$ |
| 4 | CIgreen | Chlorophyll Index Green | $\frac{nir}{green-1}$ |
| 5 | CIrededge | Chlorophyll Index RedEdge | $\frac{nir}{re-1}$ |
| 6 | CVI | Chlorophyll VI | $nir\times\frac{red}{{green}^{2}}$ |
| 7 | CI | Coloration Index | $\frac{red-blue}{red}$ |
| 8 | CTVI | Corrected Transformed VI | $\frac{NDVI+0.5}{\left\vert NDVI+0.5 \right\vert}\times\sqrt{\left\vert NDVI+0.5 \right\vert}$ |
| 9 | GDVI | Green Difference VI | $nir-green$ |
| 10 | EVI | Enhanced VI | $2.5\times\frac{nir-red}{nir+6\times red-7.5\times blue+1}$ |
| 11 | GEMI | Global Environment Monitoring Index | $a= \frac{2\times\left( {nir}^{2}-{red}^{2} \right)+1.5\times nir+0.5\times red}{nir+red+0.5}$  $GEMI=a\times\left( 1-0.25\times a \right)-\frac{red-0.125}{1-red}$ |
| 12 | GARI | Green atmospherically resistant VI | $\frac{nir-\left( green-\left( blue-red \right) \right)}{nir+\left( green+\left( blue-red \right) \right)}$ |
| 13 | GLI | Green leaf index | $\frac{2\times green-red-blue}{2\times green+red+blue}$ |
| 14 | GBNDVI | Green-Blue NDVI | $\frac{nir-\left( green+blue \right)}{nir+\left( green+blue \right)}$ |
| 15 | H | Hue | $\tan^{-1} \left( \frac{2\times red-green-blue}{30.5}\times\left( green-blue \right) \right)$ |
| 16 | MSAVI | Modified Soil Adjusted VI | $\frac{1}{2}\times\left( 2\times nir+1-\sqrt{\left( 2\times nir+1 \right)^{2}-8\times\left( nir-red \right)} \right)$ |
| 17 | NormG | Norm Green | $\frac{green}{nir+red+green}$ |
| 18 | NormNIR | Norm NIR | $\frac{nir}{nir+red+green}$ |
| 19 | NormR | Norm Red | $\frac{red}{nir+red+green}$ |
| 20 | NGRDI | Normalized green red difference index | $\frac{green-red}{green+red}$ |
| 21 | BNDVI | Blue-normalized difference VI | $\frac{nir-blue}{nir+blue}$ |
| 22 | GNDVI | Green NDVI | $\frac{nir-green}{nir+green}$ |
| 23 | NDRE | Normalized Difference Red-Edge | $\frac{nir-re}{nir+re}$ |
| 24 | RI | Redness Index | $\frac{red-green}{red+green}$ |
| 25 | NDVIrededge | Normalized Difference Rededge/Red | $\frac{re-red}{re+red}$ |
| 26 | IF | Shape Index | $\frac{2\times red-green-blue}{green-blue}$ |
| 27 | GRVI | Green Ratio VI | $\frac{nir}{green}$ |
| 28 | RRI1 | RedEdge Ratio Index 1 | $\frac{nir}{re}$ |
| 29 | IO | Iron Oxide | $\frac{red}{blue}$ |
| 30 | RGR | Red–Green Ratio | $\frac{red}{green}$ |
| 31 | SRRedNIR | Red/NIR Ratio VI | $\frac{red}{nir}$ |
| 32 | RRI2 | Rededge/Red RedEdge Ratio Index 2 | $\frac{re}{red}$ |
| 33 | TNDVI | Transformed NDVI | $\sqrt{\frac{nir-red}{nir+red}+0.5}$ |
| 34 | TGI | Triangular greenness index | $-0.5\times\left( 0.19\times\left( red-green \right)-0.12\times\left( red-blue \right) \right)$ |
| 35 | MTVI2 | Modified Triangular VI | $\frac{1.5\times\left( 1.2\times\left( nir-green \right)-2.5\times\left( red-green \right) \right)}{\sqrt{\left( 3-nir \right)^{2}-6\times nir+5\times\sqrt{red}-0.5}}$ |
| 36 | SARVI2 | Soil and Atmospherically Resistant Vegetation Index 2 | $2.5\times\frac{nir-red}{1+nir+6\times red-7.5\times blue}$ |

*VI: Vegetation index.
